# Trajectories of In-Person and Remote Care Utilization: A Sequence Analysis of Primary Care Modalities Pre- and Post-COVID-19

**DOI:** 10.21203/rs.3.rs-8176924/v1

**Published:** 2025-12-15

**Authors:** Taylor Rapson, Kathryn E Kemper-McIsaac, Namuun Clifford, Elizabeth B Sherwin, Lucia Pacca, Anna Rubinsky, Courtney Lyles, Elaine C Khoong

**Affiliations:** University of Washington; University of California San Francisco; The University of Texas at Austin; University of California San Francisco; University of California San Francisco; University of California San Francisco; University of California, Davis; University of California San Francisco

**Keywords:** Sequence analysis, health disparities, telehealth, between-visit care, healthcare utilization

## Abstract

The COVID-19 pandemic propelled the growth of virtual care, but few studies have examined how it reshaped care utilization patterns longitudinally across diverse populations. We used multichannel sequence analysis to classify 10,671 diabetic adults at two San Francisco health systems by utilization patterns from April 2019-March 2023. Patients who transitioned to digital care were Black or White, were Medicare beneficiaries, or had higher comorbidity burdens. Individuals who increased their combined in-person and virtual healthcare utilization were Hispanic with Spanish language preference and had greater disease burden. Those who decreased care utilization overall were Asian with Chinese language preference and had low patient portal engagement. While technology-enabled care models have the potential to enhance access, certain populations may be at risk of reduced access to care. Health systems should consider targeted interventions to ensure equitable access to telehealth for populations at risk of digital exclusion with the growth of remote care.

## Introduction

The COVID-19 pandemic spurred the growth of telehealth (scheduled video and/or telephone encounters) and between-visit care, such as unscheduled telephone visits and secure messaging in the patient portal, as alternative modalities for receiving healthcare^1–3^. Use of digital health and personal devices to engage in remote care has been linked to improved quality of care and clinical outcomes, particularly among individuals with chronic disease^4–9^. Evidence suggests that using the patient portal to send secure messages, request refills, and schedule appointments is linked to patient empowerment and engagement in their care, as well as improved patient-provider communication^6–12^. Similarly, use of the patient portal and telehealth has been shown to have a positive effect on self-management behaviors and clinical outcomes across chronic conditions, such as improved symptom control and medication adherence, which are critical drivers of health^4,6–8,13–16^.

Despite the well-documented increase in the use of telehealth visits and between-visit care as additional access points for engagement with primary care clinical teams in recent years, the longitudinal healthcare utilization patterns comprising multiple modalities of care and their differences across sociodemographic groups are understudied. Prior research has documented unequal uptake of digital healthcare across racial and ethnic groups, language preference, age, and gender, as well as differences in use for those facing barriers to accessing broadband internet, devices, or data (e.g., due to cost)^7,10,14,15,17–26^. In particular, older adults, especially those with non-English language preferences or who identify as a racial or ethnic minority, experience significant disparities in digital health adoption due to disparities in device access, data access, or digital literacy^8,18,22,27^. Together, these barriers create a “digital divide” that may worsen inequities in healthcare settings that increasingly rely on digital health tools, especially in systems serving low-income, diverse populations^8,14,21,28,29^. As digital health becomes further embedded in healthcare delivery, these barriers to the uptake and use of digital health tools may ultimately shape healthcare utilization and outcomes for marginalized populations^30^.

These concerns are particularly salient for patients with Type 2 Diabetes Mellitus (T2DM), one of the most common chronic conditions and one that disproportionately affects marginalized populations. Patients with uncontrolled diabetes and complications are disproportionately represented in minoritized racial and ethnic groups and lower socioeconomic neighborhoods, highlighting the need for equitable care modalities^14,31,32^. Because effective diabetes management requires ongoing monitoring, communication, and care coordination, patients with T2DM may benefit substantially from expanded modalities for accessing care. Remote tools can help address barriers to in-person care, support continuity between visits, and encourage better disease self-management. Yet, given persistent disparities in both digital health adoption and diabetes control, it remains uncertain whether the expansion of telehealth and patient portal use during the pandemic has narrowed or widened existing inequities.

Understanding healthcare utilization patterns is, therefore, critical—not only to evaluate how patients with T2DM and other chronic conditions engage with multiple modalities of care, but also to identify where gaps in care delivery may exacerbate disparities. However, most prior studies rely on repeated cross-sectional data, examining a single care modality and classifying individuals as either “high” or “low” utilizers of care^33–36^. Such designs capture snapshots rather than trajectories, limiting the ability to understand the multifaceted nature of healthcare utilization and how it evolves over time, particularly in an era of expanded digital health use^2–4^. In contrast, a longitudinal, sequence-based approach can reveal how individuals transition between in-person, telehealth, and between-visit modalities, illuminating dynamic patterns that reflect real-world care experiences. Identifying patterns of underutilization across modalities can further pinpoint unmet needs and opportunities to improve equity. For example, a recent study highlighted that when telehealth, between-visit care, and in-person care were available to patients, there were fewer differences in utilization by race and ethnicity and preferred language, suggesting that multiple options for care utilization can reduce disparities^30^. Still, research remains limited on how these alternative access points to care can alleviate barriers to care and interact over time to supplement traditional care-seeking patterns^8,33^.

Sequence analysis is a data-driven approach that compares and groups longitudinal trajectories of events, making it well-suited for evaluating healthcare utilization patterns, as it preserves the timing, order, and duration of event^37–39^. While studies often summarize utilization as aggregate counts over fixed windows, multichannel sequence analysis can reveal when and in what order patients use in-person, telehealth, and between-visit care, identifying recognizable patterns (for example, “transitioned to digital” or “reduced care engagement”^39–41^. This moves beyond simple categorizations of “high” or “low” utilizers and can surface nuanced variation in utilization patterns. The ability to cluster patients based on the timing, order, and duration of their healthcare encounters can provide a more granular understanding of patient heterogeneity and reveal hidden patterns and subgroups^42,43^.

This study advances the existing research by employing multichannel sequence analysis and cluster analysis to create clusters of patients with similar temporal sequences of in-person, between-visit, and telehealth interactions across two health systems. We examine sociodemographic differences between clusters of healthcare utilization to assess whether specific patient characteristics are disproportionately represented in certain clusters. By comparing clusters across two health systems, we aim to 1) identify heterogeneities in multi-modality healthcare utilization over time, 2) assess whether clusters of care utilization are patterned by socio-demographic factors, which could suggest potential inequities in access and utilization, and 3) assess whether care utilization patterns are consistent across health systems or vary by site, potentially reflecting differences in patient populations, resources, and care delivery models. Given the potential role of remote care engagement to either mitigate or perpetuate existing disparities, the evidence from these comparisons can shape policies that promote equitable integration of digital health across diverse healthcare settings.

## Methods

### Study Setting and Data Source

We conducted a 4-year retrospective longitudinal cohort study of adults with Type 2 Diabetes Mellitus (T2DM) receiving care at two health systems in San Francisco: the University of California, San Francisco (UCSF) and the San Francisco Health Network (SFHN). UCSF is an academic tertiary care center with four primary care practices. SFHN is an integrated, urban safety-net health system with 14 primary care practices. The study period was April 1, 2019 – March 31, 2023 at UCSF and October 1, 2019 – March 31, 2023 at SFHN. The difference in start date was due to the adoption of a new EHR software in August 2019 at SFHN.

We used electronic health records (EHR) from the two health systems, capturing sociodemographic data, clinical characteristics, and encounter data. Since the study procedures involved only a review of data collected for routine clinical care, informed consent was waived due to minimal risk to patients in the cohort. All study procedures were approved by the UCSF Institutional Review Board (IRB #20–31253) in accordance with the Declaration of Helsinki. We report our procedures and results in accordance with the STROBE guidelines.

### Study Sample

We identified a cohort of adult patients ≥ 18 years old with T2DM who were assigned a primary care provider as of April 1, 2019, at UCSF and SFHN. The creation of the cohort is described in detail in a previous study^30^ (Methodological Appendix). In brief, patients with T2DM were identified based on the following criteria before the study start date: an active DM health maintenance modifier; having a T2DM-related ICD-9/ICD-10 code on their active problem list; the most recent HbA1c test result ≥6.5%, and/or having an active diabetes medication on their medication list. To ensure active engagement in care, patients were only included if they had at least one encounter with their diabetes (primary care or endocrinology) team between April 1, 2019 – March 31, 2021, and at least one healthcare interaction (including patient portal messages, phone calls) in the last 12 months of the study period (April 1, 2022 – March 31, 2023).

### Exposure variables

We included a comprehensive set of patient-level sociodemographic characteristics as of 4/1/2019 that were extracted from the EHR. We included age (as a continuous variable and categorized as 18–34, 35–49, 50–64, 65–74, or 75+), race and ethnicity (categorized as Hispanic/Latino(a); non-Hispanic Asian; non-Hispanic Black; non-Hispanic White/Caucasian; or other non-Hispanic races, which included American Indian or Alaskan Native, Native Hawaiian or Other Pacific Islander), preferred language (English, Chinese, Spanish, or Other), sex (male/female), health insurance as of April 2019 (Commercial/Private, Medicare, Medicaid, Healthy Workers [an SF-specific program that provides health insurance coverage to people working in In-Home Supportive Services, foster care, or other publicly funded roles who do not qualify for traditional Medicaid or employer-sponsored coverage], or Uninsured), and neighborhood socioeconomic status (nSES) based on geocoded addresses, and categorized as quintiles from low to high^44^. Further details about insurance status and nSES are included in the Methodological Appendix.

Clinical characteristics included the Charlson comorbidity index (CCI, modified to exclude diabetes) from 03/01/2019–02/28/2020, baseline A1c as of 04/01/2019, and baseline blood pressure control as of 04/01/2019. We dichotomized CCI as 0–2 or 3 + to represent the severity of disease comorbidities^45^. Baseline A1c control was categorized as: controlled A1c (A1c ≤ 8), uncontrolled A1c (A1c >8), or missing. Baseline blood pressure was categorized as: ≤ 120/80 (no hypertension), >120/80 and ≤ 140/90 (controlled hypertension), or >140/90 mmHg (uncontrolled hypertension). Patient portal enrollment was included as a proxy measure of digital access. Patient portal enrollment status was extracted from the EHR as of 4/1/2020 and documented as active or inactive (yes/no).

### Construction of Care Trajectories and Cluster Analysis

We conducted sequence and cluster analysis to group patients into homogeneous clusters of healthcare utilization over time. This approach enabled us to measure the similarity of each patient’s sequence for each encounter modality across quarters and cluster similar sequences together^37^. For example, a patient whose sequence includes frequent in-person visits early in the study period followed by a shift to telehealth visits may be grouped in a cluster representing patients who transitioned from traditional to digital care modalities. To account for multiple modalities (in-person visits, telehealth visits, and between-visit interactions), we employed multichannel (also called multidimensional) sequence analysis, which allows creating three distinct channels, one for each encounter type^46^. Analyses were conducted in R Version 4.2.1 using the “TraMineR,” “cluster,” and “UniversalCVI” packages^47–50^.

#### Step 1: Building individual sequences across three channels

We summarized care utilization across three distinct types of patient encounters with the healthcare team (comprised of physicians, advanced practice providers, nurses, social workers, pharmacists, dietitians, medical assistants, or embedded behavioral health clinicians):

1. *In-person visits*: scheduled in-person visits with a member of the care team.
2. *Telehealth visits*: scheduled video or telephone visits with a member of the care team.
3. *Between-visit interactions*: care that occurred between visits, documented in the EHR as unscheduled telephone calls or patient portal messages.

For each patient, quarterly counts of encounters for each modality were grouped into ordered categories based on utilization levels. These categories were designed to capture meaningful differences in utilization while maintaining sufficient sample sizes within each category (details in Methodological Appendix). In both health systems, Q2 2020 (April–June 2020) was excluded from the analysis due to the unique encounter patterns during the COVID-19 Shelter-in-Place period (further detailed in Appendix B). For each modality, quarterly counts were binned into the following ordered categories:

1. *In-person visits*: UCSF: 0, 1, 2, 3+; SFHN: 0, 1, 2, 3–5, 6+
2. *Telehealth visits*: UCSF: 0, 1, 2+; SFHN: 0, 1, 2
3. *Between-visit interactions*: UCSF: 0,1,2,3,4–6,7+; SFHN: 0, 1, 2,3–4, 5+

#### Step 2: Sequence analysis to quantify differences between trajectories

After building the individual sequences, we then used multichannel sequence analysis to compare patients’ trajectories over time, computing differences between sequences for each modality using the dynamic Hamming distances (DHD) method^51^ (further detailed in Methodological Appendix). This approach allowed us to assess whole-person care patterns over time and identify clusters of patients with similar utilization trajectories.

#### Step 3: Cluster analysis to group similar trajectories

We grouped patients into healthcare utilization clusters using agglomerative hierarchical clustering with Ward’s linkage. Cluster quality was evaluated using indicators such as the Average Silhouette Width (ASW), the Hubert’s C index (HC), and the Calinski–Harabasz (CH) index to identify cluster solutions with high homogeneity within clusters and distinction between clusters (see Appendix C for more details). Since several cluster solutions may have similar quality, we also used expert judgement to make considerations on parsimony and salience and choose the final number of clusters^52^.

To ease our comparison of clusters within and between health systems, we conducted post-hoc analyses to categorize clusters based on similar overall trends in utilization. We grouped clusters into categories based on their trajectories after the shelter-in-place mandate was lifted, relative to their pre-pandemic behavior. We further characterized clusters by pre-pandemic in-person utilization levels.

### Statistical analyses

We described patient characteristics overall and by healthcare utilization trajectory. In addition to the exposure variables listed above, we included an interaction term between race and ethnicity and preferred language to capture the intersectional and integrated nature of these two variables. Categorical variables were compared using chi-squared tests. Analyses were conducted separately for each health system, with cross-system comparisons of care trajectories and pre-pandemic utilization presented descriptively.

### Sensitivity analyses

Sensitivity analyses were conducted to assess the robustness of the clustering approach, ensuring the stability of cluster solutions under different assumptions and methodologies. We repeated the sequence and cluster analysis using an alternative sequence dissimilarity measure and compared the resulting cluster solutions and interpretations.

## Results

First, we separately outlined the visit trajectories within each healthcare system, given their unique patient populations and different uptake of telehealth and between-visit care during and post-pandemic.

### UCSF

#### Patient Characteristics

After excluding patients without a visit in the last 12 months of the study period, we identified a cohort of 3,860 patients with T2DM at UCSF (Table 1). Over half (53%) of patients were aged 65+. Most patients were English-speaking (82%), and the cohort included high proportions of Asian (40%, including 12% with non-English preference) and White (24%) patients. Patients were relatively evenly distributed across nSES quintiles, with 17% residing in the lowest and 18% in the highest quintile. Most patients in the cohort had insurance coverage through Commercial/private (44%) and Medicare plans (43%). Almost a quarter (23%) of patients had high comorbidity scores (CCI 3+), and nearly a third had uncontrolled A1c at baseline. Digital engagement was high, with 78% of patients having an active MyChart account at baseline.

#### Description of Care Utilization Clusters

**Figure 1:**
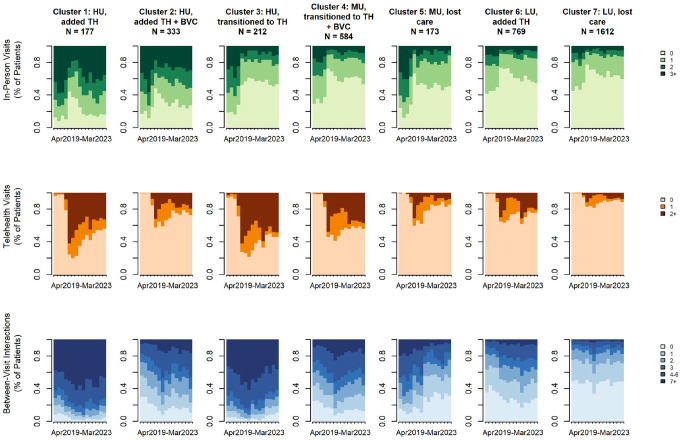
State distribution plots of In-Person, Telehealth, and Between-Visit Care Clusters at UCSF The state distribution plot shows the proportion of individuals in each care state at each time point. The x-axis represents time (e.g., months or encounters), and the stacked colored areas indicate the relative frequency of each state. Changes in the height of each colored band over time reflect shifts in the overall composition of care states across the cohort, allowing visualization of dominant modalities and temporal trends in care engagement. HU: High Utilizer, MU: Moderate Utilizer, LU: Low Utilizer, TH: Telehealth, BVC: Between-Visit Care Interactions

We identified seven distinct care utilization clusters as the final number of clusters within the UCSF diabetes cohort, representing diverse patterns of engagement with in-person, telehealth, and between-visit care modalities over time. Figure 1 displays the distribution of each care modality at each time point by cluster. Index plots showing all the individual trajectories in each cluster are displayed in Appendix E.1.

1. **High Utilizers who added Telehealth** (n = 177): These patients had very high in-person and between-visit care before the pandemic. About a year after the shelter-in-place mandate, they returned to very high in-person care and sustained high levels of between-visit care alongside moderate telehealth uptake.
2. **High utilizers who added Telehealth and Between-Visit care** (n = 333): Patients in this cluster had high in-person and moderate between-visit care before the pandemic. Following the shelter-in-place mandate, they returned to prior in-person care levels, sustained moderate increases in telehealth, and increased between-visit care.
3. **High utilizers who transitioned to Telehealth** (n = 212): These patients had high in-person, very high between-visit care, and some telehealth use before the pandemic. Post-shelter-in-place, they shifted to high telehealth and between-visit care without returning to prior in-person rates.
4. **Moderate utilizers who transitioned to Telehealth** (n = 584): These patients had moderate in-person and between-visit care before the pandemic. Post-shelter-in-place, they shifted to moderate telehealth and increased between-visit care without returning to prior in-person rates.
5. **Moderate utilizers who lost care** (n = 173): These patients had moderate in-person and between-visit care before the pandemic but did not recover either modality after the shelf-in-place period, and telehealth uptake was minimal.
6. **Low utilizers who added Telehealth** (n = 769): These patients had low in-person and between-visit care before the pandemic. Post-shelter-in-place, they returned nearly to prior in-person rates, while adding telehealth and slightly increasing between-visit care.
7. **Low utilizers who lost care** (n = 1,612): These patients had low utilization across all modalities before and after the shelter-in-place mandate. They did not return to pre-pandemic in-person rates, demonstrated minimal telehealth uptake, and had no between-visit care.

#### Sociodemographic Characteristics by Post-Pandemic Care Trajectories

Table 2 details the seven clusters classified by pre-pandemic utilization and post-pandemic changes in in-person, telehealth, and between-visit care trajectories over time. For comparisons of sociodemographic and clinical characteristics, clusters were grouped into three post-pandemic trajectories: added digital care (telehealth and/or between-visit care), transitioned to digital care (telehealth and/or between-visit care), and lost care. Among the 3,860 patients, 33% added digital care, 21% transitioned to digital care, and 46% lost care. Sociodemographic composition varied significantly across trajectories (Table 3, full descriptive tables for each cluster are presented in Appendix D.1). Patients who added digital care were more likely to be female (p = .02) and Black/African American (p < 0.001), or Hispanic with non-English language preference (p < 0.001) compared to patients who lost care or transitioned to digital care. These patients were also slightly more likely to be covered by Medicaid (p = .031), have high comorbidity scores (CCI 3+) (p < 0.001), and uncontrolled A1c at baseline (p < 0.001).

Patients who transitioned to digital care were more likely to be White, Black/African American, or Hispanic with an English-language preference (p < 0.001), and be covered by Medicaid or Medicare (p = .031) compared to patients in other care trajectories. Individuals who transitioned to digital care also had higher comorbidity scores (CCI 3+) (p < 0.001), were less likely to have missing A1c or blood pressure values (p < 0.001 and p = 0.001, respectively) and were more likely to have an active patient portal at baseline (p < 0.001). Patients who lost care were more likely to be male (p = 0.02), Asian - particularly with a non-English language preference (p < 0.001), and commercially insured (p = .031) compared to patients in other care trajectories. Patients who lost care were also more likely to have lower comorbidity scores (CCI 0–2) (p < 0.001), missing baseline A1c or blood pressure results (p < 0.001 and p = 0.001, respectively), and low patient portal enrollment (p < 0.001).

### SFHN

#### Patient Characteristics

After excluding patients without a visit in the last 12 months of the study period, we identified a cohort of 6,811 patients with diabetes at SFHN (Table 1). The SFHN diabetes cohort was younger than the UCSF cohort, with two-thirds of patients under the age of 65. Most patients at this health system were non-English speakers (57%), including 28% identifying as Hispanic and Spanish preference and 26% identifying as Asian and Chinese preference. Most patients were insured through Medicaid (40%), Medicare (31%), or Healthy Workers (15%), and nearly two-thirds (64%) resided in neighborhoods within the lowest two nSES quintiles. While 85% of patients had lower comorbidity scores (CCI < 3), 39% had uncontrolled A1c at baseline. Digital engagement was notably low, with less than 10% of patients having an active MyChart account at baseline.

#### Description of Care Utilization Clusters

**Figure 2:**
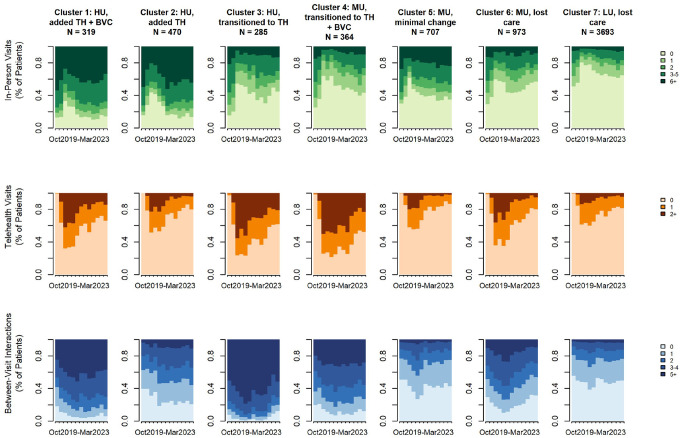
Plots of In-Person, Telehealth, and Between-Visit Care Clusters at SFHN The state distribution plot shows the proportion of individuals in each care state at each time point. The x-axis represents time (e.g., months or encounters), and the stacked colored areas indicate the relative frequency of each state. Changes in the height of each colored band over time reflect shifts in the overall composition of care states across the cohort, allowing visualization of dominant modalities and temporal trends in care engagement. HU: High Utilizer, MU: Moderate Utilizer, LU: Low Utilizer, TH: Telehealth, BVC: Between-Visit Care Interactions

We identified seven care utilization clusters as the final number of clusters within the SFHN cohort, described below. Figure 2 displays the distribution of each care modality at each time point by cluster. Index plots showing all the individual trajectories are displayed in Appendix E.2.

1. **High utilizers who added Telehealth and Between-Visit care** (n = 319): These patients had high in-person and between-visit care prior to the pandemic. Within one year of the shelter-in-place mandate, they returned to pre-pandemic in-person care levels while adding more between-visit care and moderate telehealth uptake.
2. **High utilizers who added Between-Visit care** (n = 470): These patients had high in-person and moderate between-visit care before the pandemic. A year after the shelter-in-place mandate was lifted, they returned to prior in-person rates, while increasing between-visit care. Telehealth uptake was minimal and not sustained over time.
3. **High utilizers who transitioned to Telehealth** (n = 285): These patients had high in-person and between-visit care with some telehealth use before the pandemic. Post-shelter-in-place, they did not return to pre-pandemic in-person care levels but maintained high between-visit care and had high adoption of telehealth.
4. **Moderate utilizers who transitioned to Telehealth and Between-Visit care** (n = 364): These patients had moderate in-person and high between-visit engagement prior to the pandemic. Post-shelter-in-place, they did not return to pre-pandemic in-person levels but had high telehealth uptake and increased between-visit care.
5. **Moderate utilizers with minimal change** (n = 707): These patients had moderate in-person and low between-visit care prior to the pandemic. After the shelter-in-place mandate lifted, their in-person care levels nearly returned to pre-pandemic levels, with slight telehealth uptake and between-visit engagement that was not sustained over time.
6. **Moderate utilizers who lost care** (n = 973): These patients had moderate in-person and between-visit care utilization prior to the pandemic. After the shelter-in-place mandate, they did not recover in-person care levels, which continued to decline over time, with any telehealth and between-visit care are also trending downward.
7. **Low utilizers who lost care** (n = 3,693): These patients had low in-person and between-visit utilization before the pandemic. Post-shelter-in-place, they did not return to in-person levels and maintained minimal between-visit care, with any telehealth uptake was not sustained.

#### Sociodemographic Characteristics by Post-Pandemic Care Trajectories

We classified the seven SFHN clusters by pre-pandemic utilization and post-pandemic changes in in-person, telehealth, and between-visit care (Table 4) and grouped them into four trajectories: added digital care (telehealth and/or between-visit care), transitioned to digital care (telehealth and/or between-visit care), lost care, and sustained minimal change (Table 5, full descriptive tables for each cluster are presented in Appendix D.2). Patients who added digital care were more likely to be ≥ 50 years old (p = .037), Hispanic with a non-English language preference (p < 0.001), and insured through Medicaid or Medicare (p < 0.001) compared to other care trajectories. Clinically, these patients were also more likely to have high comorbidity scores (CCI 3+) (p < 0.001) and uncontrolled A1c levels at baseline (p < 0.001). Patients who transitioned to digital care were more likely to be 75+ years old (p = .037), Black/African American or White (p < 0.001), and much less likely to have a non-English language preference (p < 0.001) compared to other care trajectories. These patients were most likely to be covered by Medicare, had high comorbidity scores (CCI 3+), have controlled baseline A1c levels, and have an active patient portal at baseline (p < 0.001 for all characteristics). Patients who lost care were more likely to be Asian - particularly with a non-English language preference (p < 0.001) and be enrolled in Healthy Workers (p < 0.001) compared to other care trajectories. Clinically, these patients were more likely to have lower comorbidity scores (CCI 0–2), missing baseline A1c and blood pressure results, and low patient portal enrollment (p < 0.001 for all characteristics). SFHN patients who had minimal change in care patterns were more likely to be 35–49 years old (p = .037), Hispanic/Latino with a non-English language preference (p < 0.001), and uninsured (p < 0.001) compared to other care trajectories. Clinically, they were also more likely to have lower comorbidity scores (CCI 0–2), slightly more likely to have uncontrolled A1c at baseline, and a very low likelihood of being enrolled in the patient portal (p < 0.001 for all characteristics).

#### Key Patterns in Care Trajectories Across Health Systems

##### Care Trajectories by Post-Pandemic Changes in Utilization

Next, we descriptively compared these trajectories across healthcare systems to identify important patterns and/or differences. UCSF saw higher proportions of patients who added or transitioned to digital care, with 33% of patients adding digital care and 21% transitioning to digital care (Table 3). In contrast, SFHN had lower proportions in these categories: 12% added digital care and 10% transitioned to digital care (Table 5). At UCSF, those who added digital care (Clusters UCSF 1, UCSF 2, UCSF 6) demonstrated sustained moderate to high telehealth uptake and varying rates of between-visit interactions, but with an increase in use overall (Fig. 1). At SFHN, however, those who added digital care (Clusters SFHN 1, SFHN 2) showed low to moderate uptake of telehealth (that was not sustained) and higher volumes of between-visit care (Fig. 2).

At both UCSF and SFHN, those who transitioned to digital care (Clusters UCSF 3, UCSF 4, SFHN 3, SFHN 4) showed moderate to high uptake of telehealth. Patients who transitioned to digital care had heterogeneous uptake of between-visit care, as some clusters increased their interactions (Clusters UCSF 4, SFHN 4) while others maintained moderate to high uptake (with no sustained increase in volume; Clusters UCSF 3, SFHN 3).

SFHN exhibited higher proportions of patients who either maintained prior care patterns with minimal changes or experienced care disengagement: 10% maintained in-person visit rates with minimal to no uptake of digital care, and 69% experienced care disruptions and lost care (compared to 46% of patients who lost care at UCSF). Patients who lost care (Clusters UCSF 5, UCSF 7, SFHN 6, SFHN 7) demonstrated low adoption of telehealth and between-visit care, often characterized by consistent non-usage or decreasing trends in use. In-person visit rates also did not recover to pre-pandemic levels among the clusters that lost care.

##### Care Trajectories by Pre-Pandemic Utilization

To compare care trajectories across both health systems, we focused on how care patterns changed for participants who were high, moderate, or low utilizers in the pre-pandemic period (see Appendix F for a detailed description). In brief, high utilizers mostly returned to their pre-pandemic in-person rates of care and incorporated both telehealth and/or between-visit care with sustained use of between-visit care modalities. Moderate utilizers had more heterogeneity, with some transitioning to digital care, some losing care, and some showing minimal changes. Low utilizers were the largest cluster in both health systems; some low utilizers lost care, while some added telehealth care.

#### Sensitivity Analysis

Sensitivity analyses using Optimal Matching (OM) instead of Dynamic Hamming Distance (DHD) produced broadly similar trajectory types. While OM yielded some variation in the distribution of patients, the overall care patterns and substantive interpretations remained consistent with the DHD results (Appendix G).

## Discussion

In a large, diverse sample of patients with T2DM across two large San Francisco health systems, we characterized clusters of multi-modal healthcare utilization patterns across three types of visits (in-person, telehealth visits, and between-visit encounters) from April 2019 to March 2023 using sequence and cluster analysis. Across both UCSF and SFHN, three distinct patterns emerged: adding digital care, where in-person visit volumes returned to baseline while telehealth and between-visit communications remained elevated; transitioning to digital care, characterized by sustained reductions in in-person visits alongside increased telehealth and/or between-visit use; and loss of care, with neither in-person nor digital services returning to pre-pandemic levels. At SFHN, a fourth pattern—minimal change—was observed, reflecting stable use of in-person visits with persistently low use of telehealth and between-visit care. Within these patterns, we noticed heterogeneity not only in the degree of telehealth uptake and sustainability of between-visit care, but also in the timing of these shifts—differences that were captured through our use of sequence analysis—highlighting nuanced variation both across and within health systems. We then examined whether sociodemographic and clinical factors were associated with distinct healthcare utilization trajectories. Our findings provide critical insights into the factors shaping transitions to digital care, sustained in-person use, and care loss.

### Post-Pandemic Shifts in Care Trajectories

A key finding was the identification of characteristics among the clusters that adopted digital care. Several clusters reflected a transition from primarily in-person care to primarily digital engagement after the shelter–in–place mandate, which was more common among Black or White patients with an English-language preference, those covered by Medicare, and those with higher comorbidity burdens. These patterns suggest that digital modalities may be particularly attractive or accessible for managing complex health needs or for those facing barriers to consistent in-person care, such as transportation, work schedules, or mobility issues, which are more pronounced among marginalized groups^53,54^. These results align with the existing literature, which indicates the value of hybrid models of care in increasing accessibility and supporting chronic disease management^4,6,7,9,29,53–55^. These findings are particularly insightful because they both corroborate prior literature on digital shifts in care delivery and extend it to a broader population, including both academic and safety-net settings. By analyzing two health systems serving demographically distinct populations, we demonstrate that digital care adoption is not limited to well-resourced populations but also emerges as a critical tool for sustaining care engagement in more diverse, underserved settings. Notably, UCSF clusters experienced a higher uptake of telehealth compared to those at SFHN, which is surprising since SFHN primarily relied on generally more accessible between-visit interactions or phone visits, whereas patients at UCSF had higher proportions of video visits^30^.

### Heterogeneity in Sociodemographic Characteristics

Race and ethnicity, preferred language, and health insurance were associated with each care trajectory, yet their distributions differed across systems. This heterogeneity implies that demographic factors do not determine care utilization but can vary depending on the context, such as the health system’s capacity or digital health outreach approach. Notably, race and ethnicity and preferred language were similar across both health systems, with patients who added digital care being more likely to be Spanish-speaking Hispanic, particularly at SFHN; patients who transitioned to digital care were more likely to be White or Black, and those who lost care were more likely to be Asian with a non-English language preference. Differences by race and ethnicity and language indicate that cultural and linguistic factors may shape how patients engage with digital care, reflecting persistent barriers as well as opportunities to enhance access. At the same time, system-level elements, such as language-concordant communication, digital literacy support, and targeted outreach, likely play a critical role in determining which patients are able to adopt and sustain digital care use, underscoring the need for equity-oriented implementation strategies^23,56,57^. The fact that individuals who added digital care were more likely to be Spanish-speaking suggests that both health systems were successful in offering and supporting the adoption of digital care options for Spanish speakers and can serve as a model for engaging diverse populations who may face barriers to digital engagement.

Moreover, there was notable heterogeneity in health insurance coverage across the two health systems, highlighting how structural context may shape care engagement. These differences reflect the broader roles of each system: UCSF as a tertiary referral center with a commercially insured base, and SFHN as a safety-net system serving populations with more limited financial and digital access. Insurance status corresponded closely to care trajectories. At UCSF, patients who lost care were more likely to be commercially insured, a group that may experience weaker continuity with a single health system due to broader care alternatives, potentially reflecting data loss rather than true change in care patterns. At SFHN, uninsured patients were overrepresented among those who lost care or exhibited minimal changes in utilization. For uninsured patients, financial barriers and limited access options likely constrain sustained engagement, while for commercially insured patients, greater flexibility in provider choice may diffuse continuity across systems. Insurance type functions as a structural determinant of healthcare engagement, influencing not only access but also the stability and modality of care over time. Health systems serving distinct payer mixes, therefore, face different equity challenges: safety-net systems must continue to address affordability and digital access, while commercially oriented systems may need to strengthen coordination and continuity within fragmented care networks. Addressing these gaps through targeted interventions—such as insurance navigation support, proactive outreach, and integrated digital care supports—may improve continuity and mitigate disparities across diverse insurance contexts. Additionally, examining how policy-level factors, such as insurance or network restrictions, influence care engagement could inform system-wide strategies to prevent care loss across diverse populations.

### Care Trajectories Based on Pre-Pandemic Utilization

When examining care trajectories through the lens of patients’ pre-pandemic utilization levels, clear trends emerged across both health systems. High utilizers prior to the pandemic were the most likely to adopt or transition to digital care, demonstrating moderate to high telehealth uptake and between-visit care engagement. This suggests that the most engaged patients were more likely to incorporate digital modalities into their care. Notably, there was one UCSF cluster of low utilizers that also added care, demonstrating some heterogeneity. No clusters among moderate utilizers showed consistent trends of adding care.

Those who transitioned to digital care tended to be moderate to high utilizers prior to the pandemic, adopting telehealth and between-visit care as a partial substitute for lost in-person visits. However, for both the trajectories that added digital care or transitioned to digital care, sustained between-visit growth was not universal, suggesting that engagement in care between visits may ebb and flow depending on patients’ needs and interests. Future studies should expand upon this work to evaluate if and how between-visit care supports care access.

Patients who lost care constituted the largest segments at both systems, mainly comprised of low or moderate pre-pandemic utilizers with limited telehealth uptake. This pattern underscores how individuals with historically lower engagement may be particularly vulnerable to care disruption and highlights the limited capacity of digital modalities alone to re-engage these patients. Notably, SFHN had a larger group of low utilizers who added care, suggesting that targeted outreach or leveraging more accessible modalities of engagement (such as greater emphasis on telephone calls versus video visits) may mitigate disengagement.

### Future Directions

These results underscore the complex interplay between digital health adoption, socioeconomic factors, and healthcare disparities in urban contexts. By examining how patients navigate and combine multiple care modalities, we can better understand how these options may create complexity, overwhelm, or mismatch with patient needs, ultimately leading to reduced engagement.

In this study, those who lost care or sustained minimal changes in their care were more likely to speak Chinese or Spanish. Several recent studies demonstrated that Chinese and Spanish speakers were disproportionately more likely to struggle with specific tasks required to use digital health tools effectively, reinforcing that linguistic and cultural barriers influence how patients access and interact with different modalities and aspects of care^18,59,65^. However, a recent study at SFHN found that language-concordant digital skills training particularly benefits Spanish speakers and patients with lower digital engagement, highlighting a possible strategy to address barriers to and reduce disparities in engagement among those at risk of care disengagement^66^. Designing tailored interventions and strategies for moderate and low utilizers at risk for care disengagement, including language-concordant support, digital literacy programs, and outreach efforts, has the potential to address barriers such as perceived usefulness and digital skills.

The differences in cluster patterns at UCSF and SFHN also indicate that digital engagement is not one-size-fits-all, and the importance of tailoring digital adoption strategies to the structural and patient-level realities of each organization, including digital preferences and needs^30,53,54^. Clusters at SFHN demonstrated moderate to low telehealth engagement, compared to UCSF, which had multiple clusters with high telehealth adoption; but SFHN patients were more likely to have sustained or increased their between-visit engagement. This highlights the continued importance of telephone calls (as opposed to patient portal messages) as an approach to engage historically excluded populations^30^.

Lastly, future studies should consider integrating clinical outcomes. Linking utilization care trajectories to longitudinal health outcomes (such as glycemic control, blood pressure, or hospitalizations), will further clarify whether digital care adoption translates into measurable health benefits, and for whom.

### Limitations

This study has some limitations. First, as this is an observational study using EHR data, we cannot rule out residual confounding, particularly related to unmeasured factors such as patient motivation, access to devices and internet, and lifestyle factors or social norms that may influence attitude towards diabetes and remote engagement in care. While we included a range of clinical and sociodemographic covariates, selection into different care modalities is non-random and likely reflects underlying health engagement, care-seeking behavior, or attitudes toward different care modalities. Inclusion in the study was restricted to those who had at least one interaction with the healthcare system both pre- and post-pandemic, and results are therefore only representative of an empaneled, care-seeking population. Lastly, results are likely not generalizable to rural settings due to differences in digital access and infrastructure.

## Conclusion

In two urban health system cohorts of adults with Type 2 diabetes analyzed over multiple years, we found that care utilization trajectories differed by sociodemographic and clinical characteristics. Specifically, in the post-pandemic era, we saw a substantial proportion of the patient population at both health systems embrace digital care (both telehealth and between-visit care), suggesting that increased digital accessibility supplements and allows patients to engage in care in ways that better suit their needs. However, a significant proportion of patients either lost access to care and experienced care disruptions or did not adopt digital care, suggesting a need for future research and tailored strategies to ensure equity in digital engagement and to close gaps in care continuity. As healthcare systems continue to expand hybrid models of care, it is critical to ensure equitable access and effectiveness across diverse populations. Future work should build upon this research and explore ways to mitigate disparities in the use of all care modalities, particularly for diverse patients and healthcare systems.

## Supplementary Material

Supplementary Files

This is a list of supplementary files associated with this preprint. Click to download.

- MethodologicalAppendix.docx

## Figures and Tables

**Table 1.** Patient Sociodemographic and Clinical Characteristics at UCSF and SFHN

	UCSF	SFHN
	n=3,860	n=6,811
**Age Category (%)**		
18–34	91 (2%)	162 (2%)
35–49	472 (12%)	1,080 (16%)
50–64	1,234 (32%)	3,215 (47%)
65–74	1,118 (29%)	1,727 (25%)
75+	945 (24%)	627 (9%)
**Sex (%)**		
Female	2,091 (54%)	3,581 (53%)
Male	1,769 (46%)	3,229 (47%)
**Joint Race Eth & Language (%)**		
Asian & English Preference	1,050 (27%)	641 (9%)
Asian & Non-English Preference	478 (12%)	1,753 (26%)
Black/African American & English Preference	527 (14%)	1,027 (15%)
Hispanic & English Preference	366 (9%)	449 (7%)
Hispanic & Non-English Preference	114 (3%)	1,881 (28%)
Other/Un & English Preference	317 (8%)	254 (4%)
Other/Un & Non-English Preference	67 (2%)	84 (1%)
White & English Preference	941 (24%)	722 (11%)
**Race or Ethnicity (%)**		
Asian	1,528 (40%)	2,394 (35%)
Black/African American	527 (14%)	1,027 (15%)
Hispanic/Latino	480 (12%)	2,330 (34%)
Other/Unknown	384 (10%)	338 (5%)
White	941 (24%)	722 (11%)
**Language Preference (%)**		
Chinese	296 (8%)	1,324 (19%)
English	3,154 (82%)	2,931 (43%)
Other	298 (8%)	655 (10%)
Spanish	112 (3%)	1,901 (28%)
**Insurance (%)**		
Commercial	1,708 (44%)	71 (1%)
Healthy Workers	0 (0%)	1,049 (15%)
Medicaid	502 (13%)	2,709 (40%)
Medicare	1,644 (43%)	2,126 (31%)
Missing	5 (0%)	28 (0%)
Uninsured	1 (0%)	828 (12%)
**Neighborhood SES (1=lowest) (%)**		
1 (lowest)	651 (17%)	2,561 (38%)
2	689 (18%)	1,771 (26%)
3	752 (19%)	1,049 (15%)
4	886 (23%)	889 (13%)
5 (highest)	698 (18%)	405 (6%)
Missing	184 (5%)	136 (2%)
**Charlson Score (%)**		
0–2	2,965 (77%)	5,818 (85%)
3+	895 (23%)	993 (15%)
**Baseline A1c Control (%)**		
Uncontrolled A1c (≥8)	818 (21%)	2,631 (39%)
Controlled A1c (<8)	2,611 (68%)	3,307 (49%)
Missing	431 (11%)	873 (13%)
**Baseline BP Control (%)**		
BP <=120/80	658 (17%)	1,629 (24%)
BP <=140/90	2,126 (55%)	3,598 (53%)
BP >140/90	930 (24%)	1,205 (18%)
Missing	146 (4%)	379 (6%)
**Active MyChart (%)**		
No	861 (22%)	6,217 (91%)
Yes	2,999 (78%)	594 (9%)

**Table 2.** Descriptions of UCSF Healthcare Trajectories by Cluster

Pre-Pandemic Utilization	Cluster	N (%)	How Each Care Pattern Changed Relative to Pre-Pandemic			Post-Pandemic Care Trajectory
			In-person	Telehealth	Between Visits	
High Utilizers	UCSF 1	177 (4.6%)	Returned to pre levels	High initial uptake, moderate sustained use	Continued high usage with slight increase overall	Added Telehealth
High Utilizers	UCSF 2	333 (8.6%)	Returned to almost pre levels	Sustained moderate uptake	Moderate uptake with slight increase overall	Added Telehealth and Between-Visit Care
High Utilizers	UCSF 3	212 (5.5%)	Did not recover to pre levels	Sustained high uptake	Continued high usage (no sustained increase)	Transitioned to Telehealth
Moderate Utilizers	UCSF 4	584 (15.1%)	Did not recover to pre levels	Sustained moderate uptake	Moderate uptake with slight increase overall	Transitioned to Telehealth and Between-Visit Care
Moderate Utilizers	UCSF 5	173 (4.5%)	Did not recover to pre levels	Moderate uptake not sustained	Moderate usage, decreasing trends	Lost Care
Low Utilizers	UCSF 6	769 (19.9%)	Returned to almost pre-levels	Sustained moderate uptake	Low uptake with increase overall	Added Telehealth
Low Utilizers	UCSF 7	1612 (41.8%)	Did not recover to pre levels	Low, consistent non usage	Low, consistent non usage	Lost Care

We defined adding digital care as in-person visit rates returning to pre-pandemic levels, while telehealth and/or between-visit care remain elevated. Those who transitioned to digital care saw in-person visit rates remain below pre-pandemic levels, while telehealth and between-visit care increased to replace in-person care. Those who lost care had minimal uptake of between-visit care or telehealth, even though in-person care rates did not rebound. We also further specified whether those who added or transitioned to digital care primarily adopted Telehealth, Between-Visit Care, or both.

**Table 3.** Sociodemographic Characteristics of Care Trajectories at UCSF

	Total N (%)	Added Digital Care	Transitioned to Digital Care	Lost Care	P-value
		Clusters 1, 2, 6	Clusters 3, 4	Clusters 5, 7	
**N (%)**	N=3,860	1,279 (33)	796 (21)	1,785 (46)	
**Age Category (%)**					0.079
18–34	91 (2)	28 (2)	22 (3)	41 (2)	
35–49	472 (12)	155 (12)	113 (14)	204 (11)	
50–64	1,234 (32)	419 (33)	267 (34)	548 (31)	
65–74	1,118 (29)	347 (27)	227 (29)	544 (30)	
75+	945 (24)	330 (26)	167 (21)	448 (25)	
**Sex (%)**					0.020
Female	2,091 (54)	726 (57)	440 (55)	925 (52)	
Male	1,769 (46)	553 (43)	356 (45)	860 (48)	
**Joint Race Ethnicity & Language (%)**					<0.001
Asian & English Preference	1,050 (27)	327 (26)	150 (19)	573 (32)	
Asian & Non-English Preference	478 (12)	145 (11)	55 (7)	278 (16)	
Black/African American	527 (14)	216 (17)	141 (18)	170 (10)	
Hispanic & English Preference	366 (9)	109 (9)	86 (11)	171 (10)	
Hispanic & Non-English Preference	114 (3)	52 (4)	17 (2)	45 (3)	
Other/Un & English Preference	317 (8)	104 (8)	66 (8)	147 (8)	
Other/Un & Non-English Preference	67 (2)	31 (2)	6 (1)	30 (2)	
White	941 (24)	295 (23)	275 (35)	371 (21)	
**Race or Ethnicity (%)**					<0.001
Asian	1,528 (40)	472 (37)	205 (26)	851 (48)	
Black/African American	527 (14)	216 (17)	141 (18)	170 (10)	
Hispanic/Latino	480 (12)	161 (13)	103 (13)	216 (12)	
Other/Unknown	384 (10)	135 (11)	72 (9)	177 (10)	
White	941 (24)	295 (23)	275 (35)	371 (21)	
**Language Preference (%)**					<0.001
Chinese	296 (8)	93 (7)	25 (3)	178 (10)	
English	3,154 (82)	1,029 (80)	714 (90)	1,411 (79)	
Other	298 (8)	105 (8)	42 (5)	151 (8)	
Spanish	112 (3)	52 (4)	15 (2)	45 (3)	
**Insurance (%)**					0.031
Commercial	1,708 (44)	559 (44)	329 (41)	820 (46)	
Medicaid	502 (13)	187 (15)	120 (15)	195 (11)	
Medicare	1,644 (43)	531 (42)	347 (44)	766 (43)	
Uninsured	1 (0)	0 (0)	0 (0)	1 (0)	
Missing	5 (0)	2 (0)	0 (0)	3 (0)	
**Neighborhood SES (1=lowest) (%)**					0.056
1	651 (17)	221 (17)	157 (20)	273 (15)	
2	689 (18)	245 (19)	144 (18)	300 (17)	
3	752 (19)	232 (18)	147 (18)	373 (21)	
4	886 (23)	304 (24)	158 (20)	424 (24)	
5	698 (18)	219 (17)	149 (19)	330 (18)	
Missing	184 (5)	58 (5)	41 (5)	85 (5)	
**Charlson Score (%)**					<0.001
0–2	2,965 (77)	939 (73)	539 (68)	1,487 (83)	
3+	895 (23)	340 (27)	257 (32)	298 (17)	
**Baseline A1c Control (%)**					<0.001
Uncontrolled A1c (>=8)	818 (21)	289 (23)	192 (24)	337 (19)	
Controlled A1c (<8)	2,611 (68)	849 (66)	549 (69)	1,213 (68)	
Missing	431 (11)	141 (11)	55 (7)	235 (13)	
**Baseline BP Control (%)**					0.001
BP <=120/80	658 (17)	220 (17)	147 (18)	291 (16)	
BP <=140/90	2,126 (55)	716 (56)	430 (54)	980 (55)	
BP >140/90	930 (24)	301 (24)	206 (26)	423 (24)	
Missing	146 (4)	42 (3)	13 (2)	91 (5)	
**Patient Portal Enrollment (%)**					<0.001
No	861 (22)	293 (23)	97 (12)	471 (26)	
Yes	2,999 (78)	986 (77)	699 (88)	1,314 (74)	
Missing					

SES: socioeconomic status, A1c: hemoglobin A1c lab result, BP: blood pressure

* P values compare patient characteristics by care trajectory based on Chi-squared test for categorical measures

**Table 4.** Descriptions of SFHN Healthcare Trajectories by Cluster

Pre-Pandemic Utilization	Cluster	N (%)	How Each Care Pattern Changed Relative to Pre-Pandemic			Post-Pandemic Care Trajectory
			In-person	Telehealth	Between Visits	
High Utilizers	SFHN 1	319 (4.7%)	Returned to pre levels	Moderate initial uptake, some sustained use	Continued high usage with slight increase overall	Added Telehealth and Between-Visit Care
High Utilizers	SFHN 2	470 (6.9%)	Returned to pre levels	Low uptake not sustained	Moderate uptake with slight increase overall	Added Between-Visit Care
High Utilizers	SFHN 3	285 (4.2%)	Did not recover to pre levels	High initial uptake, moderate sustained use	Continued high usage (no sustained increase)	Transitioned to Telehealth
Moderate Utilizers	SFHN 4	364 (5.3%)	Did not recover to pre levels	High initial uptake, moderate sustained use	Continued high usage with slight increase overall	Transitioned to Telehealth and Between-Visit Care
Moderate Utilizers	SFHN 5	707 (10.4%)	Returned to pre levels	Low uptake not sustained	Low uptake with increase overall	Minimal Change
Moderate Utilizers	SFHN 6	973 (14.3%)	Did not recover to pre levels	Moderate uptake not sustained	Moderate usage, decreasing trends	Lost Care
Low Utilizers	SFHN 7	3693 (54.2%)	Did not recover to pre levels	Low uptake not sustained	Low, consistent non usage	Lost Care

We defined adding digital care as in-person visit rates returning to pre-pandemic levels, while telehealth and/or between-visit care remain elevated. Those who transitioned to digital care saw in-person visit rates remain below pre-pandemic levels, while telehealth and between-visit care increased to replace in-person care. Those who lost care had minimal uptake of between-visit care or telehealth, even though in-person care rates did not rebound. Those with minimal change experienced minimal changes to their care utilization patterns from before the pandemic. We also further specified whether those who added or transitioned to digital care primarily adopted Telehealth, Between-Visit Care, or both.

**Table 5.** Sociodemographic Characteristics of Care Trajectories at SFHN

	Total	Added Digital Care	Transitioned to Digital Care	Lost Care	Minimal Change	P-value*
		Clusters 1,2	Clusters 3, 4	Clusters 6, 7	Cluster 5	
**N (%)**	N=6,811	N=789 (12)	N=649 (10)	N=4,666 (69)	N=707 (10)	
**Age Category (%)**						0.037
18–34	162 (2)	10 (1)	16 (2)	123 (3)	13 (2)	
35–49	1,080 (16)	118 (15)	101 (16)	723 (15)	138 (20)	
50–64	3,215 (47)	378 (48)	308 (47)	2,201 (47)	328 (46)	
65–74	1,727 (25)	201 (25)	156 (24)	1,214 (26)	156 (22)	
75+	627 (9)	82 (10)	68 (10)	405 (9)	72 (10)	
**Sex (%)**						0.094
Female	3,581 (53)	394 (50)	372 (57)	2,463 (53)	352 (50)	
Male	3,229 (47)	395 (50)	277 (43)	2,202 (47)	355 (50)	
**Joint Race Ethnicity & Language (%)**						<0.001
Asian & English Preference	641 (9)	62 (8)	62 (10)	446 (10)	71 (10)	
Asian & Non-English Preference	1,753 (26)	94 (12)	85 (13)	1,442 (31)	132 (19)	
Black/African American	1,027 (15)	116 (15)	176 (27)	659 (14)	76 (11)	
Hispanic & English Preference	449 (7)	57 (7)	64 (10)	276 (6)	52 (7)	
Hispanic & Non-English Preference	1,881 (28)	340 (43)	103 (16)	1,137 (24)	301 (43)	
Other/Un & English Preference	254 (4)	24 (3)	33 (5)	173 (4)	24 (3)	
Other/Un & Non-English Preference	84 (1)	13 (2)	5 (1)	56 (1)	10 (1)	
White	722 (11)	83 (11)	121 (19)	477 (10)	41 (6)	
**Race or Ethnicity (%)**						<0.001
Asian	2,394 (35)	156 (20)	147 (23)	1,888 (40)	203 (29)	
Black/African American	1,027 (15)	116 (15)	176 (27)	659 (14)	76 (11)	
Hispanic/Latino	2,330 (34)	397 (50)	167 (26)	1,413 (30)	353 (50)	
Other/Unknown	338 (5)	37 (5)	38 (6)	229 (5)	34 (5)	
White	722 (11)	83 (11)	121 (19)	477 (10)	41 (6)	
**Language Preference (%)**						<0.001
Chinese	1,324 (19)	52 (7)	67 (10)	1,130 (24)	75 (11)	
English	2,931 (43)	335 (42)	437 (67)	1,908 (41)	251 (36)	
Other	655 (10)	55 (7)	44 (7)	480 (10)	76 (11)	
Spanish	1,901 (28)	347 (44)	101 (16)	1,148 (25)	305 (43)	
**Insurance (%)**						<0.001
Commercial	71 (1)	6 (1)	6 (1)	53 (1)	6 (1)	
Healthy Workers	1,049 (15)	44 (6)	53 (8)	875 (19)	77 (11)	
Medicaid	2,709 (40)	364 (46)	274 (42)	1,806 (39)	265 (37)	
Medicare	2,126 (31)	283 (36)	278 (43)	1,359 (29)	206 (29)	
Uninsured	828 (12)	92 (12)	38 (6)	546 (12)	152 (21)	
Missing	28 (0)	0 (0)	0 (0)	27 (1)	1 (0)	
**Neighborhood SES (1=lowest) (%)**						0.053
1	2,561 (38)	306 (39)	261 (40)	1,728 (37)	266 (38)	
2	1,771 (26)	227 (29)	147 (23)	1,199 (26)	198 (28)	
3	1,049 (15)	122 (15)	88 (14)	737 (16)	102 (14)	
4	889 (13)	78 (10)	92 (14)	634 (14)	85 (12)	
5	405 (6)	40 (5)	48 (7)	280 (6)	37 (5)	
Missing	136 (2)	16 (2)	13 (2)	88 (2)	19 (3)	
**Charlson Score (%)**						<0.001
0–2	5,818 (85)	575 (73)	477 (73)	4,148 (89)	618 (87)	
3+	993 (15)	214 (27)	172 (27)	518 (11)	89 (13)	
**Baseline A1c Control (%)**						<0.001
Uncontrolled A1c (>=8)	2,631 (39)	376 (48)	272 (42)	1,677 (36)	306 (43)	
Controlled A1c (<8)	3,307 (49)	364 (46)	337 (52)	2,273 (49)	333 (47)	
Missing	873 (13)	49 (6)	40 (6)	716 (15)	68 (10)	
**Baseline BP Control (%)**						<0.001
BP <=120/80	1,629 (24)	198 (25)	143 (22)	1,134 (24)	154 (22)	
BP <=140/90	3,598 (53)	427 (54)	351 (54)	2,418 (52)	402 (57)	
BP >140/90	1,205 (18)	147 (19)	149 (23)	783 (17)	126 (18)	
Missing	379 (6)	17 (2)	6 (1)	331 (7)	25 (4)	
**Patient Portal Enrollment (%)**						<0.001
No	6,217 (91)	724 (92)	524 (81)	4,293 (92)	676 (96)	
Yes	594 (9)	65 (8)	125 (19)	373 (8)	31 (4)	

SES: socioeconomic status, A1c: hemoglobin A1c lab result, BP: blood pressure

* P values compare patient characteristics by care trajectory based on Chi-squared test for categorical measures
